# Pre-Parathyroidectomy PTH as an Integrated Biomarker of Glandular Remodeling and Skeletal Turnover in Secondary Hyperparathyroidism

**DOI:** 10.3390/ijms27115094

**Published:** 2026-06-04

**Authors:** Min-Tser Liao, Chia-Chao Wu, Yi-Chou Hou, Kuo-Wang Tsai, Li-Jane Shih, Kuo-Cheng Lu, Chien-Lin Lu

**Affiliations:** 1 Department of Pediatrics, Taoyuan Armed Forces General Hospital, Taoyuan 32551, Taiwan; 2 Department of Pediatrics, Tri-Service General Hospital, National Defense Medical University, Taipei 11490, Taiwan; 3 Division of Nephrology, Department of Internal Medicine, Tri-Service General Hospital, National Defense Medical University, Taipei 11490, Taiwan; 4 Division of Nephrology, Department of Internal Medicine, Cardinal-Tien Hospital, School of Medicine, College of Medicine, Fu Jen Catholic University, New Taipei City 24205, Taiwan; 5 Department of Research, Taipei Tzu Chi Hospital, Buddhist Tzu Chi Medical Foundation, New Taipei City 23142, Taiwan; 6 Department of Medical Laboratory, Taoyuan Armed Forces General Hospital, Taoyuan 32551, Taiwan; 7 Graduate Institute of Medical Science, National Defense Medical Center, Taipei 11490, Taiwan; 8 Division of Nephrology, Department of Medicine, Taipei Tzu Chi Hospital, Buddhist Tzu Chi Medical Foundation, New Taipei City 23142, Taiwan; 9 Division of Nephrology, Department of Internal Medicine, Fu Jen Catholic University Hospital, Fu Jen Catholic University, New Taipei City 24352, Taiwan; 10 School of Medicine, College of Medicine, Fu Jen Catholic University, New Taipei City 24205, Taiwan

**Keywords:** chronic kidney disease–mineral and bone disorder, secondary hyperparathyroidism, parathyroid hormone, parathyroid gland remodeling

## Abstract

Secondary hyperparathyroidism (SHPT) is a major component of chronic kidney disease–mineral and bone disorder (CKD-MBD), reflecting progressive disturbances in mineral metabolism, endocrine signaling, skeletal remodeling, and parathyroid-gland biology. Traditionally, preoperative parathyroid hormone (PTH) has been used primarily as a biochemical threshold for surgical referral. However, persistent PTH elevation in advanced CKD-related SHPT may reflect more than isolated endocrine activity; available evidence suggests it integrates parathyroid-gland remodeling, receptor resistance, skeletal turnover, treatment refractoriness, and systemic CKD-MBD severity. This review summarizes key molecular and cellular mechanisms of progressive SHPT, including diffuse-to-nodular hyperplastic transition, downregulation of calcium-sensing receptor (CaSR) and vitamin D receptor (VDR) signaling, disruption of the fibroblast growth factor 23 (FGF23)–Klotho axis, and activation of transforming growth factor-α (TGF-α)/epidermal growth factor receptor (EGFR) proliferative pathways. Building on this mechanistic framework, we discuss how persistent PTH elevation has been linked to glandular remodeling, resistance to calcimimetic and vitamin D therapy, high-turnover renal osteodystrophy, hungry bone syndrome, altered intraoperative PTH kinetics, postoperative endocrine–skeletal remodeling, and long-term recurrence. Severe SHPT is also increasingly recognized as a systemic CKD-MBD phenotype associated with vascular calcification, cardiovascular risk, metabolic instability, and impaired quality of life. Within this framework, preoperative PTH is best interpreted as an integrated biomarker within a broader assessment of glandular remodeling, skeletal metabolic activity, endocrine resistance, and systemic CKD-MBD biology, rather than as an isolated biochemical threshold.

## 1. Introduction

### 1.1. CKD-MBD and the Evolving Systems Biology of SHPT

Secondary hyperparathyroidism (SHPT) is a central component of chronic kidney disease–mineral and bone disorder (CKD-MBD), particularly as kidney function declines toward advanced CKD and dialysis dependence. CKD-MBD may begin before overt hyperphosphatemia becomes clinically apparent, with early increases in fibroblast growth factor 23 (FGF23), declining calcitriol levels, and reduced Klotho expression occurring alongside progressive phosphate retention [1]. FGF23 initially acts as an adaptive phosphaturic hormone. However, sustained FGF23 elevation suppresses renal 1α-hydroxylase activity, lowers calcitriol production, and may reduce intestinal calcium absorption, thereby contributing to progressive stimulation of parathyroid hormone (PTH) secretion [2]. As CKD advances, impaired FGF23–Klotho signaling and reduced parathyroid Klotho expression may further weaken the inhibitory effect of FGF23 on PTH secretion, favoring persistent SHPT [3].

At the glandular level, chronic hypocalcemia, hyperphosphatemia, and reduced calcitriol signaling stimulate PTH synthesis and parathyroid-cell proliferation. Early SHPT is generally characterized by diffuse polyclonal hyperplasia, whereas progressive disease may evolve toward nodular, often clonal hyperplasia with reduced calcium-sensing receptor (CaSR), vitamin D receptor (VDR), and Klotho expression [4,5]. This molecular remodeling reduces the sensitivity of the parathyroid glands to calcium-, vitamin D-, and FGF23-mediated feedback regulation, helping explain why advanced SHPT may become increasingly autonomous and less responsive to medical therapy [6]. Beyond receptor downregulation, activation of transforming growth factor-α (TGF-α)/epidermal growth factor receptor (EGFR) signaling and downstream proliferative pathways has been implicated in parathyroid-cell proliferation. Experimental CKD models further suggest that EGFR blockade or parathyroid-specific EGFR inactivation may attenuate uremia-induced parathyroid hyperplasia [7].

The clinical importance of SHPT lies in its systemic consequences within CKD-MBD. Sustained PTH excess is associated with high-turnover renal osteodystrophy, cortical bone loss, and increased fracture risk, while calcium–phosphate imbalance, elevated FGF23, and reduced vitamin D/Klotho signaling are linked to vascular and valvular calcification, cardiovascular events, and mortality in CKD populations [8]. Therefore, SHPT should not be interpreted merely as an isolated parathyroid disorder, but as an integrated manifestation of disrupted phosphate, calcium, FGF23–Klotho, vitamin D, skeletal, and vascular biology in CKD-MBD [1]. Within this framework, preoperative PTH is best understood not merely as a numerical surgical threshold, but as an integrated marker of glandular remodeling and systemic CKD-MBD burden—a concept that forms the central focus of this review.

### 1.2. The Calcimimetic Era and the Emergence of Treatment-Refractory SHPT Phenotypes

The introduction of calcimimetics, including cinacalcet, etelcalcetide, and evocalcet, has substantially changed the management of SHPT in CKD by enhancing CaSR sensitivity and suppressing PTH secretion without increasing serum calcium or phosphate levels [9]. Meta-analyses and randomized studies have shown that calcimimetics improve achievement of target PTH ranges and reduce calcium–phosphate product compared with placebo or conventional therapy, although hypocalcemia and gastrointestinal adverse effects remain important limitations [10,11]. Newer agents such as intravenous etelcalcetide and oral evocalcet may improve adherence or gastrointestinal tolerability in selected dialysis populations, but they do not fully prevent progressive glandular remodeling in advanced SHPT [12].

Importantly, the calcimimetic era has altered the timing and phenotype of patients eventually referred for parathyroidectomy. Contemporary surgical referral is generally reserved for medically refractory SHPT, often characterized by persistently elevated PTH levels (>800–1000 pg/mL), uncontrolled calcium/phosphate abnormalities, or symptomatic CKD-MBD despite maximal medical therapy [13,14]. One cohort study reported an approximately 2-year delay from SHPT diagnosis to parathyroidectomy after cinacalcet became widely available, while preoperative PTH levels were observed to remain similarly high or even increased [15]. Consequently, modern surgical candidates increasingly represent a “maximal medical therapy failure” phenotype characterized by prolonged dialysis vintage, severe biochemical derangement, larger gland burden, and advanced nodular hyperplasia [16,17].

These evolving surgical phenotypes carry important biological and perioperative implications that extend beyond biochemical thresholds alone. Patients undergoing parathyroidectomy in the calcimimetic era frequently present with markedly elevated preoperative PTH, prolonged disease duration, and severe high-turnover bone disease, all of which have been associated with increased risk of hungry bone syndrome (HBS) after surgery [18,19]. Several contemporary reviews and case series have shown that clinically significant HBS may still occur in approximately 20–70% of parathyroidectomy cohorts—a range that reflects variation in diagnostic criteria and study populations—despite prior calcimimetic exposure [18]. Accordingly, calcimimetics should not be interpreted as therapies that abolish SHPT biology, but rather as treatments that modify the temporal evolution and clinical presentation of advanced SHPT. Within this framework, persistently elevated preoperative PTH increasingly reflects cumulative glandular remodeling, skeletal turnover burden, and resistance to prolonged medical therapy instead of isolated biochemical dysregulation alone.

### 1.3. Reframing Preoperative PTH as a Systems-Level Biomarker

Traditionally, preoperative PTH in CKD-related SHPT has often been used as a biochemical threshold for surgical referral, particularly when persistent PTH elevation is accompanied by uncontrolled calcium/phosphate abnormalities or symptoms. However, available evidence supports interpreting preoperative PTH as more than a single operative trigger. Persistent PTH elevation despite maximal therapy, particularly values > 800 pg/mL for more than 6 months, has been associated with nodular hyperplasia, reduced CaSR/VDR expression, and treatment-refractory disease [14,16]. Enlarged parathyroid glands, especially volumes > 500 mm^3^ or diameters > 1 cm, further suggest nodular transformation and reduced responsiveness to calcimimetics or vitamin D therapy [20].

Preoperative PTH also reflects skeletal remodeling burden. Very high PTH levels, particularly >1000–2000 pg/mL, together with elevated alkaline phosphatase (ALP), have repeatedly been identified as predictors of severe high-turnover bone disease and HBS after parathyroidectomy [18,21]. ALP thresholds around >150–300 U/L have been incorporated into HBS risk prediction tools and machine learning models, supporting the concept that PTH should be interpreted together with bone-turnover markers rather than in isolation [18,22].

**Figure 1:**
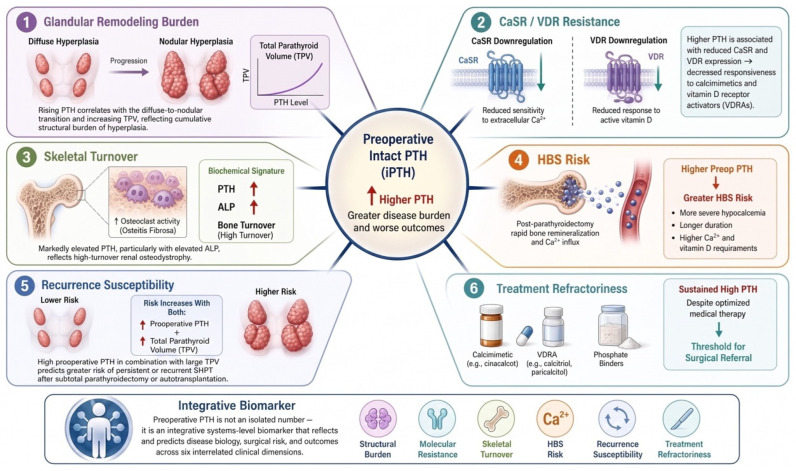
Conceptual framework illustrating preoperative intact parathyroid hormone (iPTH) as a systems-level biomarker integrating multidimensional biological and clinical manifestations of advanced secondary hyperparathyroidism (SHPT). Persistent elevation of preoperative PTH reflects cumulative glandular remodeling, calcium-sensing receptor (CaSR) and vitamin D receptor (VDR) resistance, skeletal high-turnover activity, hungry bone syndrome (HBS) susceptibility, recurrence risk, and refractoriness to medical therapy. Rather than representing an isolated biochemical threshold, preoperative PTH integrates structural, molecular, skeletal, and perioperative abnormalities across interconnected CKD-MBD pathways. Abbreviations: ALP, alkaline phosphatase; CaSR, calcium-sensing receptor; CKD-MBD, chronic kidney disease–mineral and bone disorder; HBS, hungry bone syndrome; iPTH, intact parathyroid hormone; SHPT, secondary hyperparathyroidism; TPV, total parathyroid volume; VDR, vitamin D receptor. ↑, increased.

Therefore, in the calcimimetic era, preoperative PTH is best understood as a systems-level biomarker integrating glandular remodeling, receptor resistance, skeletal turnover, treatment refractoriness, and postoperative risk, as summarized in Figure 1. Persistently elevated PTH, despite optimized calcimimetic and vitamin D therapy, is frequently regarded as an indicator of advanced, treatment-resistant SHPT and remains a common indication for parathyroidectomy [13,23]. This framework supports using preoperative PTH together with ALP, gland size, imaging findings, and clinical features to stratify disease severity and surgical risk in CKD-MBD.

This narrative review was informed by a structured search of the PubMed/MEDLINE database (last updated March 2025) using the following keywords: “secondary hyperparathyroidism,” “parathyroid hormone,” “parathyroidectomy,” “hungry bone syndrome,” “chronic kidney disease–mineral and bone disorder,” “bone turnover markers,” “alkaline phosphatase,” “calcimimetics,” and “parathyroid gland remodeling.” No formal date restriction was applied; however, priority was given to studies published within the last decade, including large cohort studies, systematic reviews, and meta-analyses. Earlier seminal works were also included to provide foundational mechanistic and clinical context. Non-English language articles were excluded. Reference lists of key articles were manually reviewed to identify additional relevant studies.

## 2. Molecular and Cellular Remodeling in Progressive SHPT

### 2.1. From Adaptive Hyperplasia to Treatment-Resistant Nodular Remodeling

**Figure 2:**
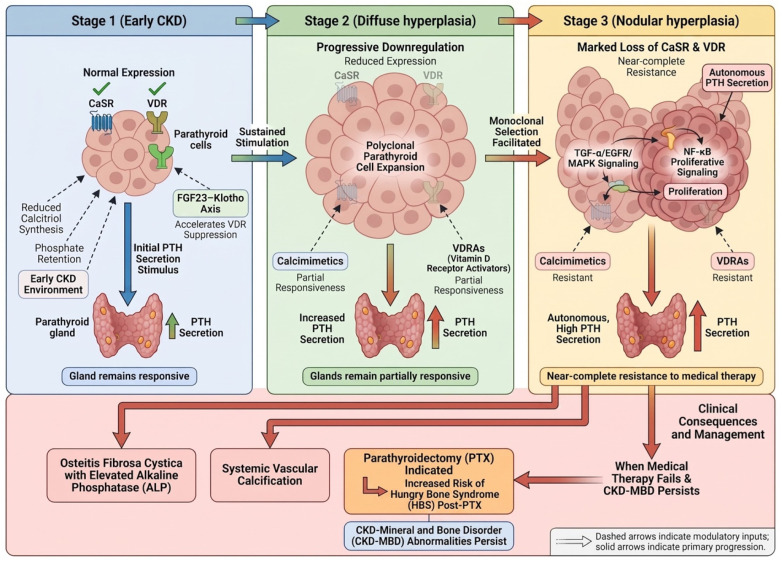
Molecular and cellular progression of secondary hyperparathyroidism (SHPT) during chronic kidney disease (CKD). Early CKD is characterized by adaptive parathyroid hormone (PTH) secretion driven by phosphate retention, calcitriol deficiency, and fibroblast growth factor 23 (FGF23)–Klotho dysregulation. Sustained stimulation promotes diffuse polyclonal hyperplasia with progressive reduction of calcium-sensing receptor (CaSR) and vitamin D receptor (VDR) expression. Advanced disease evolves toward nodular hyperplasia characterized by proliferative signaling activation, reduced responsiveness to calcimimetics and vitamin D receptor activators (VDRAs), partial endocrine autonomy, and severe CKD-mineral and bone disorder (CKD-MBD). Progressive PTH elevation reflects cumulative glandular remodeling and increasing therapeutic resistance during SHPT progression. Abbreviations: CaSR, calcium-sensing receptor; CKD, chronic kidney disease; CKD-MBD, chronic kidney disease–mineral and bone disorder; EGFR, epidermal growth factor receptor; FGF23, fibroblast growth factor 23; MAPK, mitogen-activated protein kinase; NF-κB, nuclear factor kappa B; PTH, parathyroid hormone; SHPT, secondary hyperparathyroidism; TGF-α, transforming growth factor-α; VDRA, vitamin D receptor activator; VDR, vitamin D receptor.

SHPT in CKD develops through a progressive process of parathyroid-gland remodeling driven by persistent disturbances in mineral metabolism. In the early stages of CKD, hypocalcemia, phosphate retention, reduced calcitriol synthesis, and elevated FGF23 stimulate compensatory PTH secretion and diffuse polyclonal hyperplasia of the parathyroid glands [24]. At this stage, glandular enlargement generally remains responsive to calcium-, vitamin D-, and calcimimetic-mediated feedback regulation. Experimental and pathological studies suggest that diffuse hyperplasia may still partially regress with intensive medical therapy or after kidney transplantation [25,26]. The molecular and cellular progression from adaptive diffuse hyperplasia to autonomous nodular remodeling is summarized in Figure 2.

With persistent stimulation, diffuse hyperplasia gradually progresses toward nodular remodeling characterized by monoclonal or multiclonal cellular proliferation, increased cellular density, and reduced stromal fat content [27]. Compared with diffuse hyperplasia, nodular glands exhibit lower CaSR and VDR expression, greater proliferative activity, and reduced responsiveness to medical therapy [26]. Histological studies further suggest that nodular lesions contain more oxyphil cells and show higher *Ki-67* proliferative indices, consistent with a biologically more aggressive phenotype [28]. These molecular and histological changes have direct clinical correlates. Persistent PTH elevation despite at least 6 months of optimized therapy is frequently associated with nodular hyperplasia and treatment-resistant SHPT [14,29].

Importantly, this transition from adaptive diffuse hyperplasia to nodular remodeling represents a major biological transition point in advanced SHPT. Nodular disease is associated with progressive loss of coordinated endocrine feedback regulation and increasing functional autonomy, driven in part by altered cell-cycle regulation including reduced *p21* and *p27* expression [4,30]. From a clinical standpoint, markedly elevated preoperative PTH may serve as a clinical surrogate reflecting cumulative glandular remodeling and the transition toward biologically advanced SHPT.

### 2.2. CaSR and VDR Downregulation: The Molecular Basis of Therapeutic Resistance

CaSR and VDR are central regulators of PTH secretion, PTH gene transcription, and parathyroid-cell proliferation in SHPT. CaSR senses extracellular calcium and acutely suppresses PTH secretion, whereas VDR activation by calcitriol inhibits PTH gene transcription and restrains parathyroid-cell growth [31]. During CKD progression, hypocalcemia, hyperphosphatemia, calcitriol deficiency, and uremic stress progressively impair these receptor pathways, reducing the ability of calcium and vitamin D signaling to suppress PTH secretion and glandular proliferation [32,33].

Loss of CaSR and VDR expression is most pronounced in advanced nodular SHPT. Nodular glands show marked CaSR reduction, while VDR mRNA and protein expression may be reduced by up to approximately 80% in advanced disease [34,35]. This dual receptor loss provides a mechanistic basis for therapeutic resistance: reduced CaSR limits the response to calcimimetics, whereas reduced VDR weakens calcitriol-mediated suppression of PTH transcription and parathyroid-cell proliferation [35]. Additional signaling pathways may compound this receptor-level resistance. EGFR activation may further worsen this process by lowering VDR expression and promoting parathyroid hyperplasia in experimental kidney disease models [36].

Taken together, these molecular changes translate into clinically meaningful therapeutic resistance. Progressive CaSR and VDR downregulation has been associated with reduced responsiveness to calcimimetic and vitamin D analogue therapy in advanced SHPT. Experimental data further suggest that selected VDR agonists may partially restore VDR and CaSR expression in human SHPT cells and uremic models [33].

### 2.3. FGF23–Klotho Disruption and Endocrine Mineral Dysregulation

FGF23–Klotho disruption represents an important endocrine mechanism thought to link phosphate retention, calcitriol deficiency, and progressive SHPT in CKD-MBD. In early CKD, FGF23 rises as an adaptive phosphaturic hormone to maintain phosphate balance, often before overt hyperphosphatemia or marked PTH elevation becomes apparent [1]. Physiologically, FGF23 inhibits renal 1α-hydroxylase, contributing to calcitriol deficiency, which may reduce intestinal calcium absorption and contribute to compensatory PTH stimulation [37].

As CKD progresses, renal and soluble Klotho expression decline, contributing to functional FGF23 resistance and persistent phosphate retention despite markedly elevated circulating FGF23 levels [37,38]. In advanced CKD, parathyroid Klotho and FGF receptor 1 expression may also decrease, potentially weakening the direct inhibitory effect of FGF23 on PTH secretion and thereby contributing to persistent SHPT [39]. This helps explain why very high FGF23 and PTH can coexist in kidney failure. These interconnected endocrine disturbances collectively reframe how advanced SHPT should be understood clinically. Rather than isolated parathyroid overactivity, advanced SHPT is best regarded as part of a broader endocrine network disorder involving phosphate retention, FGF23 excess, Klotho deficiency, calcitriol suppression, and impaired parathyroid feedback regulation.

### 2.4. Proliferative Signaling and Parathyroid Remodeling

Beyond mineral dysregulation, progressive SHPT is increasingly recognized as a disorder of pathologic parathyroid-cell growth signaling. Experimental CKD models and human hyperplastic or nodular glands show upregulation of TGF-α and EGFR, which is closely associated with parathyroid-cell proliferation and glandular hyperplasia [4,40]. TGF-α binding to EGFR activates downstream mitogen-activated protein kinase (MAPK) signaling, promotes *cyclin D1* expression, and drives G1-to-S cell-cycle progression, thereby supporting parathyroid-cell growth and nodular remodeling [32]. Beyond its direct proliferative effects, TGF-α/EGFR signaling may also contribute to therapeutic resistance by linking proliferative remodeling to loss of vitamin D responsiveness. In experimental SHPT, EGFR activation has been shown to reduce VDR expression and promotes calcitriol resistance, whereas EGFR inhibition or parathyroid-specific EGFR inactivation has been reported to preserve VDR expression and attenuate uremia-induced parathyroid hyperplasia [7,36]. While TGF-α/EGFR represents the most extensively studied proliferative pathway in SHPT, other pathways, including mammalian target of rapamycin (mTOR)/protein kinase B (AKT) and nuclear factor kappa B (NF-κB), have also been implicated in parathyroid-cell proliferation, particularly in nodular disease [4,41,42].

These converging proliferative mechanisms collectively reframe our understanding of advanced SHPT. Rather than solely an adaptive endocrine response to phosphate retention, hypocalcemia, and calcitriol deficiency, advanced SHPT is increasingly recognized as a progressive proliferative remodeling disorder of the parathyroid glands. This structural and molecular remodeling may help explain why nodular SHPT tends to persist despite aggressive pharmacologic suppression.

## 3. Preoperative PTH as a Biomarker of Glandular Remodeling and Treatment Refractoriness

### 3.1. Preoperative PTH and Glandular Remodeling Burden

Among patients with CKD-related SHPT, preoperative PTH reflects not only biochemical severity but also the underlying burden of parathyroid-gland remodeling. In a large surgical series of 1500 patients with SHPT, nodular hyperplasia was present in 96.5% of resected glands, and the probability of nodular hyperplasia increased with higher PTH levels and longer dialysis duration [43]. Pathophysiologic reviews also describe SHPT progression from diffuse to nodular hyperplasia, in which persistent stimulation is associated with reduced feedback responsiveness and increasing resistance to vitamin D and calcimimetic therapy [44]. While biochemical markers such as PTH provide important prognostic information, they do not fully characterize glandular anatomy or disease extent.

Preoperative imaging provides complementary information about gland burden. In CKD-related SHPT, ultrasonography, computed tomography (CT), and sestamibi scintigraphy can localize enlarged hyperplastic glands, although sensitivity varies according to gland size and functional activity [45]. Combined imaging approaches may improve detection of multiple enlarged glands, particularly in patients with multiglandular disease or reoperation settings [45]. Therefore, PTH should be interpreted alongside gland size and imaging findings rather than as an isolated marker. Acknowledging both the utility and limitations of PTH as a single parameter, markedly elevated preoperative PTH may serve as a practical clinical surrogate for advanced glandular remodeling, especially when accompanied by enlarged glands on imaging or prolonged dialysis vintage. However, PTH does not perfectly quantify total gland burden in every patient; evidence from primary hyperparathyroidism suggests only weak correlations between preoperative biochemical severity and total gland volume or multigland disease [46]. Thus, a more reliable assessment of gland burden is likely to benefit from integrating PTH, imaging, dialysis duration, biochemical control, and clinical refractoriness.

### 3.2. Preoperative Imaging in CKD-Related SHPT: Modality Performance and Selection

Preoperative imaging in CKD-related SHPT serves primarily to localize enlarged hyperplastic glands and identify ectopic or supernumerary tissue, though performance varies substantially by modality and disease burden. Ultrasound is widely used as a first-line tool given its accessibility and lack of radiation; surgeon-performed ultrasound in renal hyperparathyroidism has demonstrated sensitivity of approximately 81% but low specificity (~30%), with accuracy further reduced in the presence of ectopic glands, glands smaller than 1 cm, or concomitant goiter [47]. Sestamibi scintigraphy combined with SPECT/CT shows sensitivity of approximately 66–67% and specificity of 55–57% in end-stage renal disease patients with predominantly secondary or tertiary hyperparathyroidism, and performance deteriorates further in multiglandular disease, where sensitivity may fall below 40% [48,49].

More advanced cross-sectional and nuclear imaging modalities offer improved performance, particularly in multiglandular disease. Four-dimensional CT (4D CT) has emerged as a superior alternative, demonstrating sensitivity of approximately 89% and specificity of 64% in SHPT, and consistently outperforming sestamibi in multiglandular disease settings [50] MRI has shown comparable or slightly higher sensitivity (~91%) in SHPT, with a combined MRI plus 4D CT approach achieving a positive predictive value of approximately 97% [50]. ^18^F-fluorocholine PET/CT appears to represent the most sensitive currently available modality, with pooled sensitivity of approximately 90–97% and specificity of approximately 94% across meta-analyses of hyperparathyroidism cohorts, with sensitivity substantially exceeding that of sestamibi (0.96 versus 0.54 in direct comparison) and greater per-patient sensitivity compared with 4D CT (92% versus 85%) in meta-analyses [51,52]. Although dedicated data in pure secondary hyperparathyroidism remain limited, ^18^F-fluorocholine PET/CT has been described as the most sensitive option for multiglandular and tertiary disease where available, though cost and availability may constrain its routine use [53,54].

Integrating the performance characteristics described above, a practical imaging strategy may be guided by PTH level and gland burden. In patients with PTH 800–1000 pg/mL and glands smaller than 500 mm^3^, ultrasound may represent a reasonable initial assessment. As PTH rises above 1000–2000 pg/mL with gland enlargement exceeding 500 mm^3^, SPECT/CT or 4D CT may be preferred given the higher likelihood of multiglandular disease and the recognized limitations of ultrasound and sestamibi alone in this setting. In patients with PTH exceeding 2000 pg/mL, suspected ectopic glands, or planned reoperation, 4D CT or ^18^F-fluorocholine PET/CT should be considered, recognizing that cost and availability may limit access to the latter in routine practice.

### 3.3. PTH and Resistance to Medical Therapy

Persistent elevation of preoperative PTH despite optimized medical therapy is frequently regarded as an indicator of treatment resistance in advanced SHPT rather than inadequate dose escalation alone. Calcimimetics suppress PTH primarily through allosteric activation of CaSR, whereas active vitamin D analogues inhibit PTH gene transcription through VDR-mediated signaling [13,14]. As SHPT progresses from diffuse to nodular hyperplasia, CaSR and VDR expression decline, reducing responsiveness to both calcimimetics and vitamin D-based therapy [14]. These molecular changes are accompanied by structural glandular alterations that further predict medical refractoriness. Larger nodular glands, particularly those >500 mm^3^ or >1 cm, are associated with poorer response to pharmacologic therapy and are commonly considered markers of advanced SHPT [55]. Available evidence also suggests that parathyroid-gland volume correlates with required calcimimetic dose over time; patients with gland volume > 500 mm^3^ may require substantially higher cinacalcet-equivalent doses while maintaining higher PTH levels [20,56].

Translating these molecular and structural observations into clinical practice, persistent PTH > 800 pg/mL for more than 6 months despite maximally tolerated vitamin D analogues and calcimimetics is widely used as a marker of likely nodular or monoclonal hyperplasia and as a reason to consider parathyroidectomy [13,14]. Thus, persistently elevated preoperative PTH despite optimized therapy is best interpreted as a possible indicator of cumulative glandular remodeling and receptor-level resistance, especially when accompanied by enlarged glands or persistent calcium/phosphate abnormalities.

### 3.4. The “Maximal Medical Therapy Failure” Phenotype

The widespread use of calcimimetics has shifted SHPT management toward longer periods of medical therapy before parathyroidectomy. After calcimimetics became available, surgery has increasingly been reserved for drug-refractory SHPT, commonly characterized by persistent PTH > 800–1000 pg/mL together with uncontrolled calcium/phosphate abnormalities, symptoms, or calcification-related complications [14,18]. In one cohort, the median interval from hyperparathyroidism diagnosis to parathyroidectomy increased from 27 to 49 months after calcimimetic introduction, suggesting an approximately 22-month delay in surgical referral [15]. Another contemporary cohort reported that cinacalcet use among surgical candidates increased from 9.5% in an earlier cohort to 93.8% after 2009 [16].

This progressive delay in surgical referral, combined with longer exposure to high PTH levels, has given rise to a contemporary “maximal medical therapy failure” phenotype. These patients often have prolonged high-PTH exposure, nodular gland remodeling, and reduced responsiveness to conventional therapy rather than simply undertreated biochemical SHPT. Persistent PTH > 800–1000 pg/mL for more than 6 months despite maximally tolerated vitamin D analogues and calcimimetics suggests nodular hyperplasia with reduced receptor-mediated feedback and relative autonomy [14,57]. Recent pathology data also support the concept that long-standing nodular SHPT may acquire clonal or epigenetic features, such as *p16* promoter hypermethylation, consistent with progressive structural remodeling [28].

These biological characteristics translate into measurable perioperative implications. Longer dialysis vintage, high preoperative PTH, elevated ALP, and greater gland weight have been associated with increased postoperative HBS risk after parathyroidectomy [19]. Accordingly, preoperative PTH is best understood not only as a surgical threshold; rather, it is most informative when integrated with PTH trajectory, gland size and imaging, ALP or bone-specific ALP, symptoms, and calcification burden when determining the timing of parathyroidectomy in CKD-related SHPT.

## 4. Skeletal Remodeling and High-Turnover Bone Disease

### 4.1. PTH-Driven Skeletal Remodeling in High-Turnover Renal Osteodystrophy

Persistent PTH elevation in CKD-related SHPT has been identified as a major contributor to high-turnover renal osteodystrophy. Mechanistically, PTH acts on osteoblasts and osteocytes to increase receptor activator of nuclear factor κB ligand (RANKL), thereby promoting osteoclastogenesis and accelerated bone resorption [58]. In severe SHPT, bone multicellular unit activation frequency may rise markedly, and osteoclast surfaces have been reported to reach 10–20 times normal levels, producing extensive resorption, peritrabecular fibrosis, and abundant unmineralized osteoid [59].

Among the skeletal compartments affected, cortical bone appears particularly vulnerable to the effects of chronic PTH excess. The skeletal effects of chronic PTH excess are particularly prominent in cortical bone. CKD cohorts have shown rapid cortical deterioration, including approximately 3% per year declines in cortical area and thickness and approximately 4% per year increases in cortical porosity at the radius, with these changes associated with higher time-averaged PTH and bone-turnover markers [60]. Advanced SHPT is consequently often characterized by high-turnover osteitis fibrosa, cortical thinning, intracortical porosity, and progressive skeletal fragility [39]. These abnormalities help explain why bone quality and microarchitecture, rather than bone mineral density alone, are central to fracture risk in CKD-MBD.

Beyond its local skeletal consequences, chronic high-turnover remodeling also has systemic implications for mineral homeostasis. Sustained PTH-driven bone resorption has been associated with mobilization of skeletal calcium and phosphate, which may contribute to mineral instability in advanced CKD-MBD [18,61]. In this context, markedly elevated preoperative PTH is best interpreted not only as a marker of endocrine disease severity, but also as an indirect indicator of cumulative skeletal remodeling burden and postoperative metabolic vulnerability.

### 4.2. Bone Turnover Biomarkers and Skeletal Remodeling Burden

Although PTH remains the principal endocrine driver of high-turnover bone disease in CKD-related SHPT, bone turnover markers provide more direct information on skeletal remodeling activity. Bone-specific alkaline phosphatase (BALP) is released by osteoblasts during mineralization and correlates with bone formation rate and bone biopsy findings in CKD-MBD [62,63]. Compared with PTH, BALP and total ALP are less affected by renal clearance and may show lower biological variability, making them useful complementary markers for evaluating high-turnover versus low-turnover states [64].

Complementing these formation markers, markers of bone resorption provide additional information on osteoclastic activity and skeletal calcium efflux. Markers of bone resorption provide additional information on osteoclastic activity and skeletal calcium efflux. Tartrate-resistant acid phosphatase-5b (TRACP-5b) is produced by osteoclasts, correlates with osteoclast number and histologic resorption indices, and is less influenced by kidney function than collagen degradation markers [63]. By contrast, C-terminal telopeptide of type I collagen (CTX) reflects collagen breakdown but is partly renally cleared, which limits specificity in advanced CKD [65,66].

Given the complementary strengths and limitations of individual markers, an integrated approach may offer more reliable characterization of skeletal remodeling burden Combining PTH with BALP, ALP, TRACP-5b, serum calcium, phosphate, and imaging findings can better characterize skeletal remodeling burden than endocrine measurements alone. Studies combining PTH, BALP, procollagen type I N-terminal propeptide (P1NP), and TRACP-5b have reported diagnostic accuracy around an area under the curve of 0.8 for distinguishing low, normal, and high bone turnover compared with bone biopsy [63]. Therefore, in advanced SHPT, preoperative PTH is best interpreted within a broader remodeling profile to estimate skeletal burden and postoperative metabolic vulnerability.

### 4.3. Hungry Bone Syndrome as a Rebound Skeletal Phenomenon

**Figure 3:**
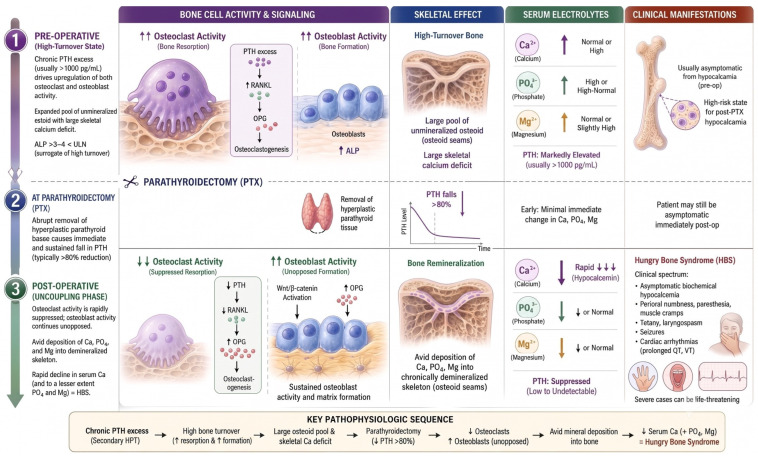
Pathophysiological sequence of hungry bone syndrome (HBS) following parathyroidectomy (PTX) in severe secondary hyperparathyroidism (SHPT). Chronic preoperative parathyroid hormone (PTH) excess promotes high-turnover bone disease characterized by accelerated osteoclastic and osteoblastic activity, expanded osteoid burden, and sustained skeletal calcium efflux. Following abrupt postoperative PTH reduction, osteoclast-mediated resorption rapidly decreases whereas osteoblast-driven mineralization remains relatively preserved, resulting in avid skeletal uptake of calcium, phosphate, and magnesium. This uncoupled remodeling state contributes to postoperative hypocalcemia, mineral shifts, and the clinical manifestations of HBS. Severe preoperative hyperparathyroidism and elevated alkaline phosphatase (ALP) reflect greater skeletal remodeling burden and increased postoperative metabolic vulnerability. Abbreviations: ALP, alkaline phosphatase; Ca, calcium; HBS, hungry bone syndrome; Mg, magnesium; OPG, osteoprotegerin; PO4, phosphate; PTH, parathyroid hormone; PTX, parathyroidectomy; RANKL, receptor activator of nuclear factor κB ligand; SHPT, secondary hyperparathyroidism. ↑, increased; ↓, decreased.

HBS after parathyroidectomy for severe SHPT is best understood as a rebound skeletal mineral-uptake phenomenon rather than a simple postoperative calcium-deficiency state, as illustrated in Figure 3. In advanced SHPT, chronic PTH excess drives high-turnover bone disease, with active osteoclasts and osteoblasts, expanded unmineralized osteoid, and sustained calcium efflux from bone into the circulation [18,67]. After parathyroidectomy, abrupt PTH reduction has been shown to suppress osteoclast-mediated resorption, while osteoblast-driven mineralization may remain elevated, potentially producing rapid influx of calcium, phosphate, and magnesium into bone [18,19].

This rebound model is supported by perioperative bone-marker patterns. Formation markers such as ALP, BALP, and osteocalcin often remain elevated or rise after surgery, whereas resorption markers such as TRACP-5b and CTX tend to fall, consistent with uncoupled remodeling favoring skeletal mineral deposition [67,68]. Clinically, hypocalcemia often begins within 12–72 h after surgery, with calcium nadirs commonly occurring around postoperative days 2–7, while ALP may peak approximately 1–2 weeks later, reflecting ongoing remineralization [19,69].

Preoperative PTH and bone-turnover markers are therefore useful surrogates of HBS risk. Very high PTH, elevated ALP or bone-specific ALP, increased TRACP-5b/CTX, low calcium, and severe bone disease have been repeatedly associated with deeper or more prolonged postoperative hypocalcemia [18]. Recent risk scores, nomograms, and machine-learning models incorporate PTH, ALP, and gland or skeletal burden to estimate postoperative HBS risk [22,68]. Thus, preoperative PTH is best interpreted not only as a marker of endocrine activity, but also as an indirect indicator of cumulative skeletal remodeling load and postoperative metabolic vulnerability.

Building on this framework, several validated multivariate prediction tools have been developed for HBS risk stratification, and models incorporating ALP consistently outperform PTH-alone approaches. The NYU 2-point score assigns one point each for ALP > 150 U/L and PTH > 1000 pg/mL; a score of 0 yields 100% negative predictive value (effectively ruling out HBS), while a score of 1 carries 93.8% positive predictive value and a score of 2 confers 100% positive predictive value, with overall accuracy of 96.8% [70]. The Gao nomogram incorporates continuous values of PTH and ALP, achieving a C-index of 0.943; notably, ALP alone demonstrated an AUC of 0.926 compared with 0.873 for PTH alone, underscoring the incremental predictive value of bone formation markers over PTH in isolation [71]. The Wang nomogram further integrates bone-specific ALP, corrected calcium, and total resected gland weight, with an overall AUC of approximately 0.92 substantially exceeding that of PTH alone (~0.84) [68].

More recently, machine learning approaches have been applied to further improve predictive accuracy. The XGBoost machine learning model incorporates PTH, ALP, corrected calcium, age, and the intraoperative PTH decline percentage (%PTH), achieving an AUC of 0.878, with a web-based calculator publicly available at https://chaiyalin.shinyapps.io/make_web/ (accessed on 31 May 2026) [22]. Across all four models, ALP-inclusive tools consistently outperform PTH-only approaches, supporting the interpretation of preoperative PTH as a necessary but insufficient standalone predictor of postoperative HBS risk.

Despite its widespread use in HBS risk prediction, total ALP is a non-specific marker with isoenzymes distributed across bone, liver, intestine, and placenta. Elevated total ALP may therefore reflect conditions unrelated to skeletal remodeling, including hepatobiliary disease, bone metastases, and the post-fracture healing period, all of which may lead to overestimation of HBS risk in affected patients. BALP, which is produced by osteoblasts, provides a more direct assessment of bone formation and skeletal turnover. In a multicenter study of 492 patients with CKD stage 5D who underwent bone biopsy, BALP demonstrated moderate diagnostic performance for distinguishing different turnover states, with area-under-the-curve values generally ranging from approximately 0.75 to 0.85 depending on the histomorphometric classification used [72]. Nevertheless, several practical limitations remain. In hemodialysis patients with chronic liver disease, liver-derived ALP may affect the interpretation of BALP measurements and reduce their specificity for bone turnover [73]. Collectively, current evidence suggests that BALP may provide a more bone-focused assessment than total ALP in CKD-MBD, although assay accessibility and comorbid liver disease should be considered when interpreting results.

## 5. Endocrine–Skeletal Remodeling Dynamics After Parathyroidectomy

### 5.1. Altered Intraoperative PTH Kinetics in CKD-Related SHPT

Intraoperative PTH (ioPTH) monitoring during parathyroidectomy for CKD-related SHPT is best interpreted within the context of altered endocrine kinetics and impaired renal peptide clearance rather than by directly extrapolating criteria developed for primary hyperparathyroidism. Intact PTH has a circulating half-life of approximately 5 min, whereas C-terminal PTH fragments may persist substantially longer and are cleared predominantly through the kidney [73]. Consequently, patients with advanced CKD exhibit delayed postoperative PTH decay kinetics, with one study reporting an estimated ioPTH half-life of 6.6 min in renal hyperparathyroidism compared with 2.2 min in patients with preserved renal function [74].

Impaired renal clearance, however, is not the only factor shaping ioPTH kinetics in SHPT. Glandular burden and systemic endocrine remodeling also contribute to the complexity of intraoperative PTH interpretation. Patients with markedly elevated preoperative PTH frequently harbor multiglandular nodular hyperplasia and large circulating pools of biologically less active C-terminal PTH fragments, both of which may prolong apparent biochemical decay after gland excision [75,76]. Accordingly, conventional criteria such as the Miami criterion (>50% reduction at 10 min) may inadequately reflect operative success in CKD-related SHPT. Several studies have therefore proposed extended monitoring intervals and stricter decline thresholds, including >80% reduction at 20 min or >90% reduction at 30 min [75,77].

Taken together, these considerations suggest that ioPTH monitoring in CKD-related SHPT is best understood as a disease-specific kinetics problem rather than simply a binary indicator of operative completeness, with markedly elevated preoperative PTH predicting greater kinetic complexity and supporting the use of prolonged monitoring intervals in biologically advanced disease [75].

### 5.2. Preoperative PTH, Glandular Phenotype, and Recurrence Susceptibility

In advanced SHPT, operative strategy is influenced not only by biochemical severity but also by the glandular phenotype reflected by preoperative PTH elevation. CKD-related SHPT is generally thought to progress from diffuse polyclonal hyperplasia to nodular, partially autonomous glands with reduced CaSR and VDR expression, which has been associated with decreased responsiveness to physiological feedback and medical therapy [24,44]. Persistent PTH elevation despite medical therapy, particularly PTH > 800 pg/mL, is commonly used as a clinical indicator of severe or refractory SHPT and may suggest nodular proliferation with receptor downregulation [13,14]. In addition, recent surgical data indicate that higher preoperative PTH and larger total parathyroid volume independently predict recurrence after parathyroidectomy, supporting the interpretation of preoperative PTH as a marker of gland burden and recurrence susceptibility rather than a purely biochemical cutoff [78].

Several cohort studies have provided more granular data on PTH-associated recurrence thresholds. One cohort identified PTH > 2000 pg/mL as an independent predictor of reintervention after surgery, whereas another study demonstrated that preoperative PTH > 928 pg/mL together with larger total parathyroid volume independently predicted recurrence after total parathyroidectomy with autotransplantation (TPTX + AT) [18,79]. These findings support the concept that persistent postoperative disease frequently reflects ongoing biological remodeling rather than isolated technical failure.

Central to understanding this recurrence biology is the concept of remnant tissue remodeling. Residual tissue preserved during subtotal parathyroidectomy or implanted during TPTX + AT retains proliferative and molecular characteristics of the original hyperplastic glands [13,80]. Nodular graft tissue may therefore continue to undergo progressive remodeling under persistent CKD-MBD stimulation, contributing to delayed recurrence over long-term follow-up [81]. Accordingly, operative decision-making is likely to be most informed by integrating preoperative PTH together with gland size, imaging characteristics, skeletal remodeling burden, calcium/phosphate abnormalities, and anticipated HBS risk rather than relying on isolated biochemical thresholds alone.

### 5.3. Postoperative Endocrine–Skeletal Remodeling Trajectories

Postoperative biochemical evolution after parathyroidectomy in CKD-related SHPT is best understood as a reflection of dynamic adaptation between residual endocrine activity, skeletal remodeling, and systemic CKD-MBD burden, rather than simple normalization of laboratory abnormalities. In successful surgery, PTH commonly declines rapidly from markedly elevated preoperative concentrations, frequently ranging from 1000 to 3000 pg/mL, to very low postoperative levels [18,82]. Early postoperative PTH reduction may also provide prognostic information, as reductions < 80% have been associated with persistent hyperparathyroidism, whereas reductions > 95% may predict more durable biochemical control [82].

While PTH kinetics provide early prognostic information, calcium dynamics in the postoperative period are governed by a distinct but related set of factors. Postoperative calcium kinetics are strongly influenced by preexisting skeletal remodeling intensity. Patients with severe high-turnover bone disease and markedly elevated preoperative PTH or ALP possess a metabolically active skeleton with substantial mineral demand. Following abrupt withdrawal of chronic PTH stimulation, persistent osteoblastic activity may promote rapid calcium influx into bone and has been associated with precipitation of HBS in susceptible patients [18,67]. Contemporary studies report HBS incidence ranging from approximately 20–70%, although variability is strongly influenced by differing diagnostic definitions and patient populations [14,19].

Beyond calcium and PTH nadirs, the interpretation of persistently elevated postoperative PTH also requires careful contextual consideration. Early inadequate PTH decline may reflect residual, ectopic, or supernumerary glands, whereas delayed biochemical recurrence may result from remnant or autograft hyperplasia [13]. Conversely, eucalcemic PTH elevation after surgery may also reflect skeletal recovery, vitamin D deficiency, or persistent CKD-MBD adaptation rather than immediate operative failure [18]. Therefore, postoperative biochemical trajectories are best interpreted as systems-level endocrine–skeletal remodeling patterns shaped by preoperative glandular burden, bone-turnover activity, and long-term CKD-MBD biology rather than isolated postoperative laboratory fluctuations.

## 6. Long-Term Outcomes and Systems-Level Prognostic Implications

### 6.1. Recurrence Biology and Remnant Gland Remodeling

Although parathyroidectomy can markedly improve biochemical control in advanced CKD-related SHPT, persistent or recurrent disease remains a long-term concern. Recurrence is best understood not only as incomplete excision; available evidence suggests it may also reflect continued remodeling of residual or autotransplanted parathyroid tissue under ongoing CKD-MBD stimulation. Nodular hyperplasia, higher proliferative activity, and *Ki-67* > 1.5% have been associated with greater recurrence risk after surgery [83,84]. The pathways through which recurrence develops, however, differ depending on the surgical approach employed.

The mechanism of recurrence differs by surgical approach. After subtotal parathyroidectomy, recurrence commonly reflects remnant hyperplasia, missed glands, or supernumerary parathyroid tissue [83]. After TPTX + AT, recurrent SHPT often arises from hyperplastic autografted tissue, with graft-dependent recurrence reported in approximately 5–24% of patients in graft-focused series [79,85]. Nodular grafts appear more likely to recur than diffuse grafts, with one study reporting recurrence rates of 24% versus 8.4%, respectively [86].

Regardless of surgical approach, accumulating evidence suggests that recurrence is driven more by underlying glandular biology than by technical factors alone. Several factors suggest that recurrence reflects aggressive glandular biology rather than a purely technical event. Very high preoperative PTH has been associated with greater gland mass, nodular remodeling, and recurrence susceptibility [14,84]. Graft-dependent recurrence often develops slowly over years, with reported median intervals of approximately 5–9 years, supporting a model of progressive graft or remnant hyperplasia rather than abrupt relapse [23,79]. Accordingly, postoperative recurrence in CKD-related SHPT is best understood as a continuation of systemic and local glandular remodeling, influenced by residual tissue volume, nodular histology, and ongoing phosphate or uremic stress.

### 6.2. Cardiovascular and Systemic Implications

Markedly elevated preoperative PTH is best interpreted as a marker of systemic CKD-MBD burden rather than isolated parathyroid-gland dysfunction. CKD-MBD is characterized by hyperphosphatemia, SHPT, and vascular calcification, all of which are strongly linked to cardiovascular morbidity and mortality [3,87]. Elevated PTH has been associated with increased bone resorption and higher circulating calcium–phosphate burden, while high FGF23 has been associated with left ventricular hypertrophy, arrhythmia, endothelial dysfunction, and cardiovascular events [88].

These mechanistic associations are reflected in observational clinical outcome data. In refractory SHPT, parathyroidectomy has been associated with improved biochemical control and lower mortality in several cohort studies. A large meta-analysis including 24,398 patients reported that parathyroidectomy was associated with approximately 50% lower all-cause mortality and reduced cardiovascular mortality compared with medical therapy, particularly in patients with higher PTH levels [89]. However, because these data are largely observational, causality should be interpreted cautiously.

Beyond cardiovascular and metabolic outcomes, severe SHPT also carries a substantial symptom burden with direct implications for quality of life. Common symptoms include bone pain, pruritus, fatigue, muscle weakness, sleep disturbance, and depressed mood, all of which may improve after parathyroidectomy in selected patients [90]. Therefore, markedly elevated preoperative PTH may serve as an integrated indicator of cardiovascular, metabolic, skeletal, and patient-reported disease burden within CKD-MBD.

### 6.3. Beyond Surgical Thresholds: Integrated Risk Stratification

**Figure 4:**
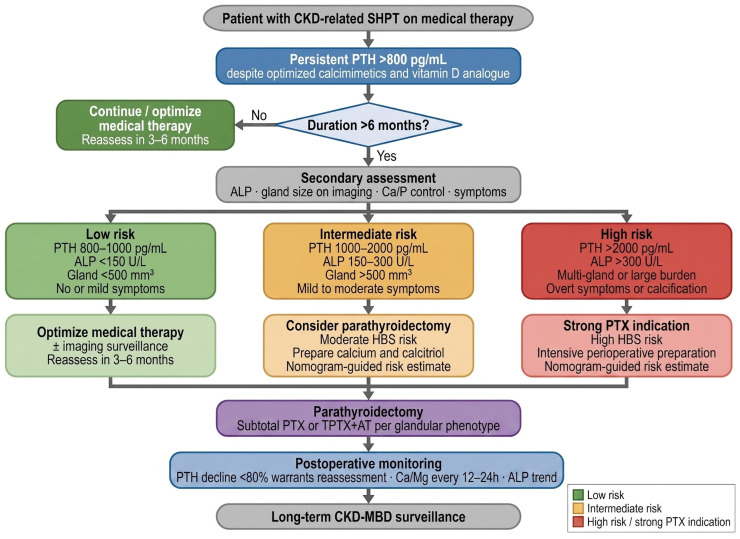
Integrated clinical decision framework for preoperative parathyroid hormone interpretation in chronic kidney disease–related secondary hyperparathyroidism. Patients with persistent PTH > 800 pg/mL despite optimized calcimimetics and vitamin D analogue therapy for more than 6 months undergo secondary assessment incorporating ALP, gland size on imaging, calcium/phosphate control, and symptom burden. Risk stratification into low (green), intermediate (orange), and high (red) categories guides the decision to continue medical therapy, consider parathyroidectomy, or proceed with strong surgical indication, respectively. Postoperative monitoring includes assessment of PTH decline, serial calcium and magnesium measurements, and ALP trend to guide calcium supplementation tapering and detect persistent disease. PTH thresholds reflect those most consistently reported across the literature and are intended as clinical guidance rather than absolute cutoffs. Abbreviations: ALP, alkaline phosphatase; Ca, calcium; CKD-MBD, chronic kidney disease–mineral and bone disorder; HBS, hungry bone syndrome; Mg, magnesium; PTH, parathyroid hormone; PTX, parathyroidectomy; SHPT, secondary hyperparathyroidism; TPTX + AT, total parathyroidectomy with autotransplantation.

The multidimensional biological and clinical implications of preoperative PTH elevation in CKD-related SHPT are summarized in Table 1. Historically, surgical referral for CKD-related SHPT has often relied on relatively fixed biochemical thresholds, commonly persistent PTH > 800–1000 pg/mL together with hypercalcemia, hyperphosphatemia, symptoms, or calcification-related complications [13,14]. However, PTH alone is an imperfect guide because it is biologically variable, influenced by assay differences and skeletal responsiveness, and may not fully reflect the complexity of systemic CKD-MBD burden. Recent observational data further suggest that combined interpretation of PTH together with ALP and other markers of skeletal turnover may provide more clinically meaningful prognostic information than isolated PTH values alone [91] (Figure 4).

Building on this evidence, a multiparametric approach integrating PTH with mineral, skeletal, structural, and symptom-based indicators offer a more comprehensive basis for surgical decision-making. Serum calcium and phosphate reflect mineral dysregulation, while ALP and BALP help identify high-turnover bone disease and HBS risk; in several studies, ALP/BALP performed well as predictors of bone turnover and postoperative hypocalcemia [18,67]. Imaging findings, including ultrasound, computed tomography, and single-photon emission computed tomography/computed tomography, provide additional information on gland burden and localization, although sensitivity varies across patients [13,45].

Taken together, these biochemical, skeletal, and imaging parameters collectively argue for a shift away from reliance on PTH thresholds alone. Contemporary decision-making is therefore served by multiparametric risk stratification that integrates PTH trajectory, calcium/phosphate control, ALP/BALP and other bone markers, gland size, imaging findings, dialysis vintage, symptoms, calcification burden, and quality-of-life impairment. Emerging nomograms and risk models incorporating PTH, ALP/BALP, calcium, dialysis duration, age, and gland weight further support this approach for predicting HBS and guiding perioperative intensity [18,71]. In this context, preoperative PTH is best understood as one central component of a broader CKD-MBD risk profile rather than as a stand-alone operative cutoff.

## 7. Future Directions

Future management of CKD-related SHPT will likely evolve from static biochemical threshold–based assessment toward longitudinal and multidimensional disease phenotyping. Although PTH remains a central indicator of disease severity, growing evidence suggests that no single biomarker adequately captures the full spectrum of glandular remodeling, skeletal turnover, endocrine resistance, and systemic CKD-MBD burden. Emerging strategies increasingly favor integrated models incorporating PTH, calcium, phosphate, ALP/BALP, FGF23, bone-turnover markers, gland volume, imaging characteristics, and clinical symptom burden to improve risk stratification and surgical decision-making [1,18]. Longitudinal assessment may be particularly important, as dynamic changes in PTH trajectory, gland enlargement, skeletal turnover, and treatment responsiveness may better reflect progression toward nodular autonomy and medical refractoriness than isolated laboratory measurements alone.

Complementing this longitudinal biochemical approach, advances in imaging and molecular phenotyping may further refine the characterization of biologically aggressive SHPT. Conventional ultrasonography and sestamibi scintigraphy remain standard localization tools, whereas newer modalities such as four-dimensional computed tomography and positron emission tomography may provide additional insight into glandular vascularity, metabolic activity, and ectopic tissue burden [13,23]. Artificial intelligence-assisted imaging analysis, radiomics, and quantitative glandular assessment also represent emerging areas of interest. These approaches may help identify imaging signatures associated with nodular remodeling, altered receptor expression, proliferative activity, and recurrence susceptibility. In parallel, molecular profiling of parathyroid tissue—including proliferative signaling pathways, receptor-expression patterns, epigenetic alterations, and remodeling signatures—may improve differentiation between medically responsive diffuse hyperplasia and biologically autonomous nodular disease.

Individualized risk stratification represents a particularly important future research priority. Current HBS prediction models, including nomograms and machine learning algorithms such as the XGBoost-based calculator, incorporate biochemical markers and limited clinical variables but do not yet integrate imaging-derived parameters such as total parathyroid volume, glandular vascularity, or metabolic activity on PET/CT. Combining these imaging biomarkers with PTH trajectory, bone turnover markers, and patient-level factors within AI-assisted predictive frameworks may substantially improve perioperative risk prediction beyond what any single parameter can achieve. Radiomics-based analysis of parathyroid gland characteristics and deep learning models trained on multimodal data represent emerging methodologies that warrant prospective validation in this population. Genetic polymorphisms in the VDR, CaSR, and components of the RANKL/OPG and Wnt pathways may further modulate individual susceptibility to HBS and recurrence, and their integration into future risk models merits dedicated investigation.

If validated prospectively, these technological and molecular advances could collectively support a broader transition toward individualized timing of parathyroidectomy, moving away from reliance on universal operative thresholds. Future decision-making will likely integrate glandular remodeling severity, skeletal turnover burden, imaging-derived gland characteristics, vascular calcification, treatment responsiveness, dialysis vintage, frailty, and postoperative metabolic vulnerability alongside conventional biochemical parameters [8,16]. Patients with rapidly enlarging glands, persistently elevated bone-turnover markers, progressive calcification, or declining calcimimetic responsiveness may benefit from earlier intervention before irreversible autonomy and severe skeletal complications develop. Conversely, excessive biochemical suppression or extensive parathyroid tissue removal may increase the risk of low-turnover bone disease and impaired skeletal adaptation. Ultimately, the goal is to transition from population-level threshold-based decisions toward patient-specific risk profiles that integrate glandular remodeling biology, skeletal metabolic activity, imaging phenotype, and treatment history to guide both the timing of surgery and the intensity of postoperative management.

## 8. Conclusions

HPT in CKD represents a progressive disorder of endocrine dysregulation, parathyroid-gland remodeling, skeletal turnover abnormalities, and systemic CKD-MBD. In the contemporary calcimimetic era, preoperative PTH is best understood not solely as a biochemical threshold for surgical referral. Rather, persistent PTH elevation has been increasingly recognized as reflecting cumulative glandular remodeling, CaSR and VDR resistance, skeletal high-turnover activity, treatment refractoriness, postoperative HBS susceptibility, and recurrence biology. Progressive SHPT is increasingly characterized by diffuse-to-nodular hyperplastic transition, activation of proliferative signaling pathways, altered endocrine responsiveness, and broader systemic complications involving vascular calcification, cardiovascular instability, and impaired quality of life.

Current evidence suggests that preoperative PTH may serve as an integrated biomarker linking molecular remodeling, skeletal metabolic activity, perioperative risk, and long-term disease behavior. Accordingly, interpretation of preoperative PTH is most informative when integrated with glandular imaging, biochemical parameters, skeletal turnover markers, and overall CKD-MBD severity, rather than when applied as an isolated biochemical cutoff in surgical decision-making. Translating this framework into clinical practice requires a shift from static threshold-based assessment toward individualized, multiparametric risk stratification that can better capture the full biological complexity of advanced CKD-related SHPT.

## Figures and Tables

**Table 1:** Systems-level implications of preoperative PTH elevation in CKD-related secondary hyperparathyroidism.

Preoperative PTH Phenotype	Biological Interpretation and Remodeling Features	Clinical Implications	HBS Risk Estimate
Persistent PTH > 800 pg/mL for >6 months despite optimized calcimimetics and vitamin D analogue	Progressive endocrine resistance with diffuse-to-nodular hyperplastic transition; reduced CaSR/VDR expression; declining responsiveness to medical therapy	PTX may be considered when biochemical and systemic CKD-MBD abnormalities persist despite maximal tolerated therapy	Low-moderate risk; HBS risk influenced by skeletal turnover markers, calcium status, and gland burden [13,14,18]
PTH > 800–1000 pg/mL with persistent hypercalcemia or hyperphosphatemia	Severe CKD-MBD mineral dysregulation phenotype; persistent phosphate retention; refractory metabolic imbalance	Earlier PTX consideration in patients with progressive vascular calcification, calciphylaxis risk, or erythropoietin-resistant anemia	Low-moderate risk; skeletal turnover burden remains variable [13,14]
PTH > 1000–2000 pg/mL with markedly elevated ALP (150–300 U/L)	High-turnover skeletal remodeling phenotype; osteitis fibrosa; accelerated bone remodeling; expanded osteoid burden; increased postoperative mineral demand	Increased susceptibility to postoperative HBS and severe calcium shifts; intensive calcium and calcitriol supplementation frequently required	Moderate-high risk; NYU 2-point score (ALP > 150 + PTH > 1000): NPV 100% (score = 0); PPV 93.8% (score = 1) and 100% (score = 2); sensitivity 100%, specificity 94.1% [70]; Wang nomogram AUC~0.92 versus PTH alone ~0.84 [68]; Gao nomogram C-index 0.943 [71]
PTH > 2000 pg/mL with severe endocrine resistance and ALP > 300 U/L	Advanced treatment-resistant nodular hyperplasia; markedly reduced CaSR/VDR expression, partial secretory autonomy; severe skeletal turnover activation	PTX strongly considered; prolonged medical suppression progressively ineffective; intensive periperative metabolic monitoring required	High risk; very high PTH and ALP levels consistently associated with severe HBS phenotypes; XGBoost model AUC 0.878; web calculator available at https://chaiyalin.shinyapps.io/make_web/ (accessed on 31 May 2026) [22].
PTH > 928 pg/mL with TPV > 1.99 cm^3^	Recurrence-prone remodeling phenotype; large gland burden; persistent proliferative potential, nodular histology	Greater risk of recurrent SHPT after subtotal PTX or TPTX + AT; recurrence reflects ongoing biological remodeling rather than isolated technical failure	Moderate–high recurrence risk; HBS-specific prediction not independently established [78]
Post-PTX PTH decline < 80% or persistent postoperative elevation	Persistent endocrine activity suggesting residual hyperfunctioning tissue, ectopic glands, supernumerary glands, or remnant hyperplasia	Persistent biochemical activity may indicate continued SHPT biology; relative PTH decline more informative than isolated postoperative values in dialysis patients	Variable; primarily predicts persistent disease rather than HBS [13,82]

Abbreviations: ALP, alkaline phosphatase; CaSR, calcium-sensing receptor; CKD-MBD, chronic kidney disease–mineral and bone disorder; HBS, hungry bone syndrome; PTH, parathyroid hormone; PTX, parathyroidectomy; SHPT, secondary hyperparathyroidism; TPV, total parathyroid volume; TPTX + AT, total parathyroidectomy with autotransplantation; VDR, vitamin D receptor.

## Data Availability

No new data were created or analyzed in this study. Data sharing is not applicable to this article.

## Abbreviations

The following abbreviations are used in this manuscript:

AKT	Protein kinase B
ALP	Alkaline phosphatase
BALP	Bone-specific alkaline phosphatase
CaSR	Calcium-sensing receptor
CKD	Chronic kidney disease
CKD-MBD	Chronic kidney disease–mineral and bone disorder
CTX	C-terminal telopeptide of type I collagen
EGFR	Epidermal growth factor receptor
FGF23	Fibroblast growth factor 23
HBS	Hungry bone syndrome
ioPTH	Intraoperative parathyroid hormone
MAPK	Mitogen-activated protein kinase
mTOR	Mammalian target of rapamycin
NF-κB	Nuclear factor kappa B
OPG	Osteoprotegerin
P1NP	Procollagen type I N-terminal propeptide
PTH	Parathyroid hormone
PTX	Parathyroidectomy
RANKL	Receptor activator of nuclear factor κB ligand
SHPT	Secondary hyperparathyroidism
TGF-α	Transforming growth factor-α
TPV	Total parathyroid volume
TRACP-5b	Tartrate-resistant acid phosphatase-5b
TPTX + AT	Total parathyroidectomy with autotransplantation
VDRA	Vitamin D receptor activator
VDR	Vitamin D receptor
